# Supplementary material for: Transfer learning of GW Bethe–Salpeter equation excitation energies
Source: Chem Sci. 2026 Feb 23;17(16):8090–9. doi: 10.1039/d5sc09780k (PMC12951312; doi:10.1039/d5sc09780k)
Supplement: SC-017-D5SC09780K-s001 [file SC-017-D5SC09780K-s001.pdf]

Supplementary Information (SI) for Chemical Science.
This journal is © The Royal Society of Chemistry 2026

Supporting Information

to

Transfer learning of GW Bethe–Salpeter Equation excitation energies

Dario Baum, Arno Förster, and Lucas Visscher*

Department of Chemistry and Pharmaceutical Sciences, Vrije Universiteit Amsterdam, De Boelelaan 1108, 1081 HZ Amsterdam, The Netherlands

E-mail: l.visscher@vu.nl

Datasets

To avoid contamination of test sets with molecules from the pretraining or finetuning sets, we applied SMILES-based duplicate removal between each pretraining dataset (OMol25 subset and QCDGE) and each test set. For models finetuned on qs*GW* QP energies, which use only OMol25 pretraining, test molecules overlapping with OMol25 were removed. For models finetuned on qs*GW*-BSE excitation energies, which use both OMol25 and QCDGE, any molecules overlapping with either pretraining dataset were removed. An identical check was performed between the finetuning dataset (QM9GWBSE) and all test sets and no SMILES matches were found. Note, that this leads to different numbers of samples in the test sets for qs*GW* QP energies and qs*GW*-BSE excitation energies. In the following, we list the specifications for all pretraining, finetuning and test sets used in this work.

Table S1: Composition of pretraining, finetuning and test sets used for training and testing qs*GW* QP energy models.

Dataset	Purpose	N_{samples}	$N_{\text{atoms}}^{\text{max}}$	Elements
OMol25	pretraining	10 000 000	176	H, Li, B, C, N, O, F, Na, Mg, Si, P, S, Cl, K, Ca, Br, I
OMol25	pretraining	5 000 000	176	H, Li, B, C, N, O, F, Na, Mg, Si, P, S, Cl, K, Ca, Br, I
OMol25	pretraining	1 000 000	176	H, Li, B, C, N, O, F, Na, Mg, Si, P, S, Cl, K, Ca, Br, I
QM9GWBSE	finetuning	120 000	29	H, C, N, O, F
QM9GWBSE	test	2658	29	H, C, N, O, F
PC9	test	1984	29	H, C, N, O, F
OE62L	test	879	48	H, C, N, O, F
OE62H	test	574	30	H, B, C, N, O, F, Si, P, S, Cl, As, Se, Br, I

Table S2: Composition of pretraining, finetuning and test sets used for training and testing qs*GW*-BSE excitation energy models.

Dataset	Purpose	N_{samples}	$N_{\text{atoms}}^{\text{max}}$	Elements
OMol25	pretraining	5 000 000	176	H, Li, B, C, N, O, F, Na, Mg, Si, P, S, Cl, K, Ca, Br, I
OMol25	pretraining	500 000	30	H, C, N, O, F
QCDGE	pretraining	400 000	32	H, C, N, O, F
QM9GWBSE	finetuning	120 000	29	H, C, N, O, F
QM9GWBSE	test	455	29	H, C, N, O, F
PC9	test	258	29	H, C, N, O, F
OE62L	test	836	48	H, C, N, O, F
OE62H	test	574	30	H, B, C, N, O, F, Si, P, S, Cl, As, Se, Br, I

Machine learning

For training ViSNet models, small ones with 889 730 parameters and large ones with 2 494 586 parameters, we use the official open-source implementation provided in the `AI2BMD` GitHub repository. The hyperparameters we used for small and large models throughout this work are listed in Tab. S3. In Tab. S4 to S12 we list all training parameters used in this work for the respective training modes, properties and model sizes.

Table S3: Hyperparameters of small and large ViSNet models.

Parameter	Value
Number of interaction layers	3
Cutoff distance	5.0
Number of hidden channels	128 (small), 216 (large)
Radial basis functions	Gaussian
Number of radial basis functions	25
Radial basis functions trainable	False
Max. angular degree	2
Activation function	SiLU
Attention activation function	SiLU
Number of heads	4
Max. number of neighbors per atom	100
Max. nuclear charge	83
Vector norm	None
Vector norm trainable	False

Table S4: Training parameters for pretraining small models on DFT MO energies, gaps and TDDFT gaps.

Parameter	Value
Batch size	256
Number of epochs	50
Number of warm-up epochs	5
Max. learning rate	0.0005
Min. learning rate	0.00005
Weight decay	0.01
Loss	Huber

Table S5: Training parameters for pretraining large models on DFT HOMO energies.

Parameter	Value
Batch size	256
Number of epochs	50
Number of warm-up epochs	5
Max. learning rate	0.00025
Min. learning rate	0.000075
Weight decay	0.01
Loss	Huber

Table S6: Training parameters for finetuning small and large models on qs*GW* QP HOMO and LUMO energies.

Parameter	Value
Batch size	256
Number of epochs	50
Number of warm-up epochs	5
Max. learning rate	0.0005
Min. learning rate	0.0001
Weight decay	0.01
Loss	MSE

Table S7: Training parameters for finetuning small models on qsGW QP gaps.

Parameter	Value
Batch size	256
Number of epochs	75
Number of warm-up epochs	5
Max. learning rate	0.0005
Min. learning rate	0.0001
Weight decay	0.01
Loss	MSE

Table S8: Training parameters for finetuning small models on qsGW-BSE excitation energies.

Parameter	Value
Batch size	256
Number of epochs	50
Number of warm-up epochs	5
Max. learning rate	0.0005
Min. learning rate	0.0001
Weight decay	0.001
Loss	MSE

Table S9: Training parameters for training small models from scratch, thus without pretraining, on qsGW HOMO and LUMO energies.

Parameter	Value
Batch size	256
Number of epochs	50
Number of warm-up epochs	5
Max. learning rate	0.0005
Min. learning rate	0.0001
Weight decay	0.01
Loss	Huber

Table S10: Training parameters for training large models from scratch, thus without pretraining, on qsGW QP HOMO energies.

Parameter	Value
Batch size	128
Number of epochs	75
Number of warm-up epochs	5
Max. learning rate	0.0002
Min. learning rate	0.0001
Weight decay	0.01
Loss	Huber

Table S11: Training parameters for training small models from scratch, thus without pretraining, on qsGW QP gaps.

Parameter	Value
Batch size	256
Number of epochs	75
Number of warm-up epochs	5
Max. learning rate	0.0005
Min. learning rate	0.0001
Weight decay	0.001
Loss	Huber

Table S12: Training parameters for training small models from scratch, thus without pretraining, on qsGW-BSE excitation energies.

Parameter	Value
Batch size	256
Number of epochs	50
Number of warm-up epochs	5
Max. learning rate	0.0005
Min. learning rate	0.0001
Weight decay	0.001
Loss	Huber

Improvement of generalization

Below, we show the test errors of small models pretrained on DFT LUMO energies or DFT gaps and subsequently finetuned on qs*GW* QP LUMO energies or qs*GW* QP gaps, respectively, using transfer learning. For comparison, we also include results from baseline models trained directly on the target property without pretraining (“Baseline”) and from models with pretraining only and no finetuning (“None”). Figure S1 presents the results for LUMO energies, and Fig. S2 shows the corresponding results for gaps.

As discussed in the main text, the findings closely mirror those for QP HOMO energy predictions. Pretraining followed by finetuning consistently lowers the test errors, with the largest improvements observed for the PC9, OE62L, and OE62H test sets. Moreover, the performance of the model without finetuning lies closer to that of the fully pretrained and finetuned model on the challenging OE62L and OE62H sets. Interestingly, in these cases the additional benefit gained from finetuning is even larger than what was observed for the QP HOMO predictions in the main text.

Consistent with the analysis in the main text, Figs. S3 and S4 display the absolute per-sample errors with and without pretraining for qs*GW* QP LUMO energies and QP gaps, respectively. As noted earlier, the same qualitative trends appear here:

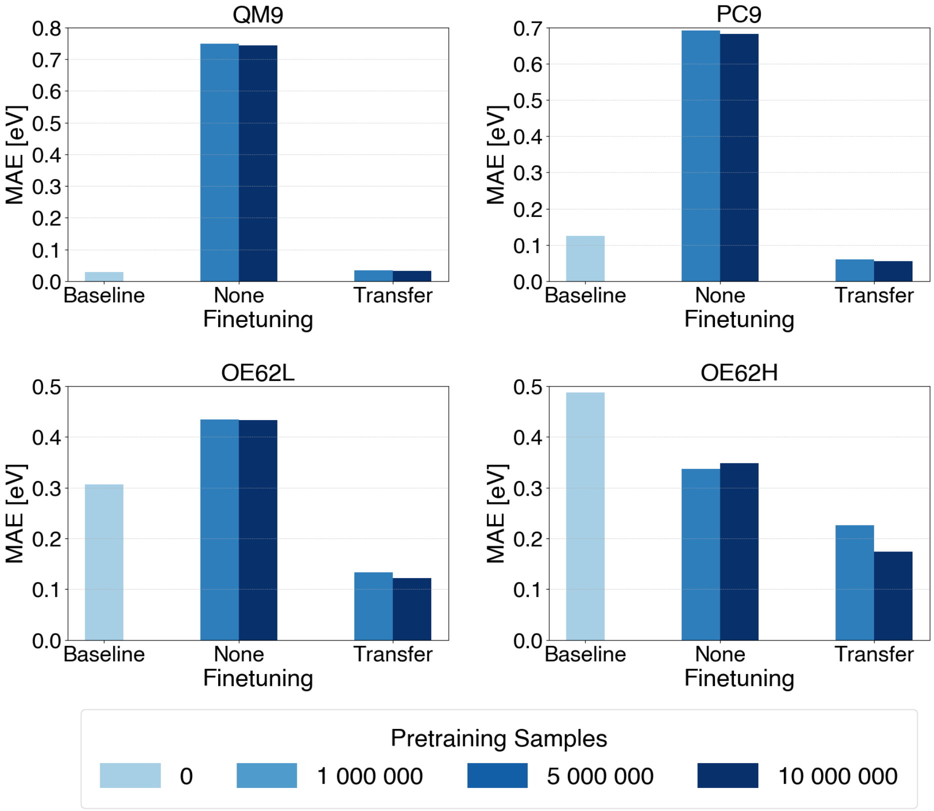

Figure S1: MAE of qs*GW* QP LUMO energy predictions from small models with different numbers of pretraining samples.

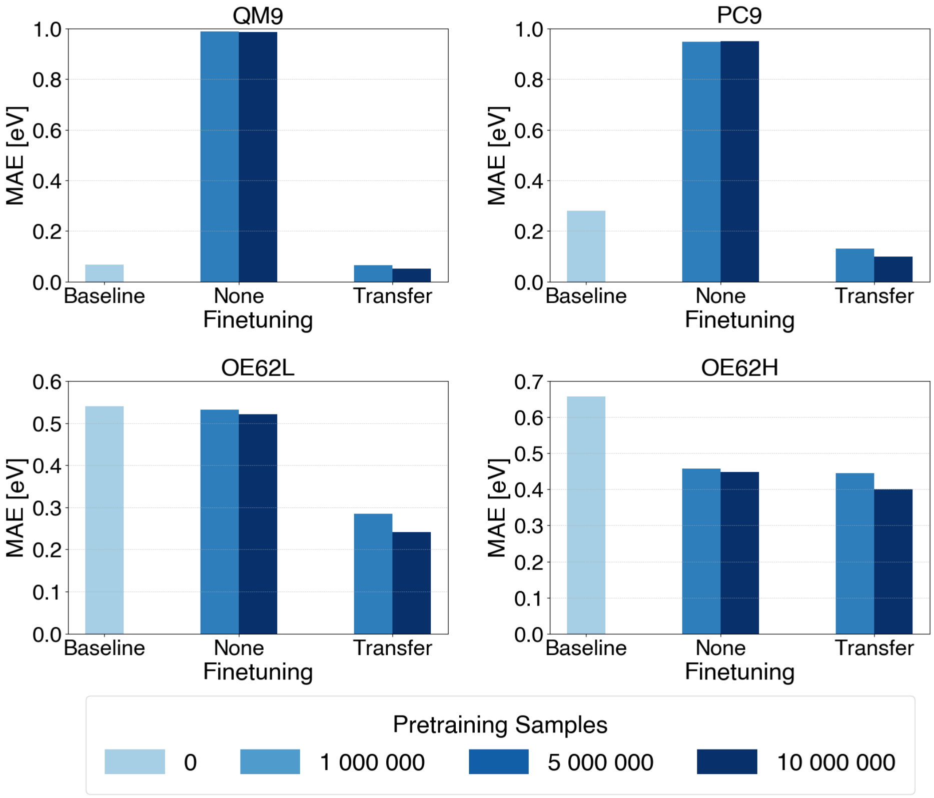

Figure S2: MAE of qs*GW* QP gap predictions from small models with different numbers of pretraining samples.

pretraining tends to reduce errors especially for samples that would otherwise be outliers when training from scratch. This effect is most pronounced for OE62L and OE62H, where a large fraction of samples show high error without pretraining, explaining the substantial overall improvement on these sets. Conversely, for the QM9 test set, where test errors of the baseline model are already low, the gains from pretraining are minimal. As also seen before, these improvements come at the cost of slightly increasing the errors for samples that already exhibit low error without pretraining, including a few severe outliers on the OE62H test set for both QP LUMO energies and QP gaps.

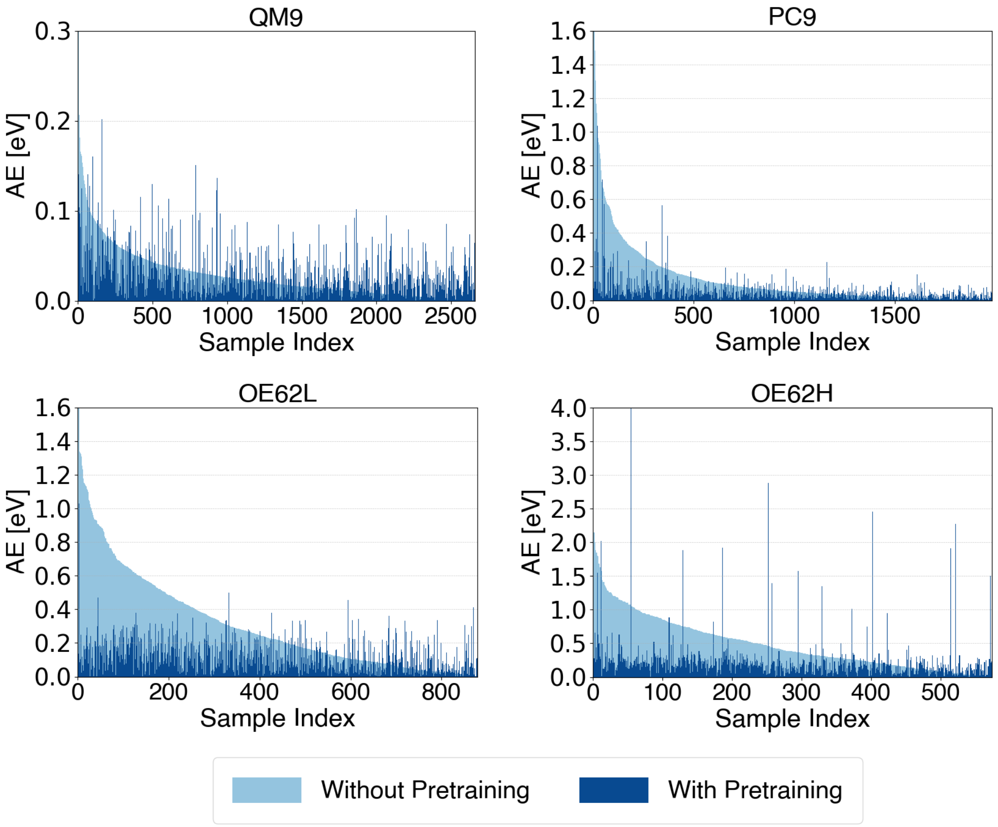

Figure S3: Per-sample absolute errors (AEs) of qs*GW* QP LUMO energy predictions from small models with and without pretraining.

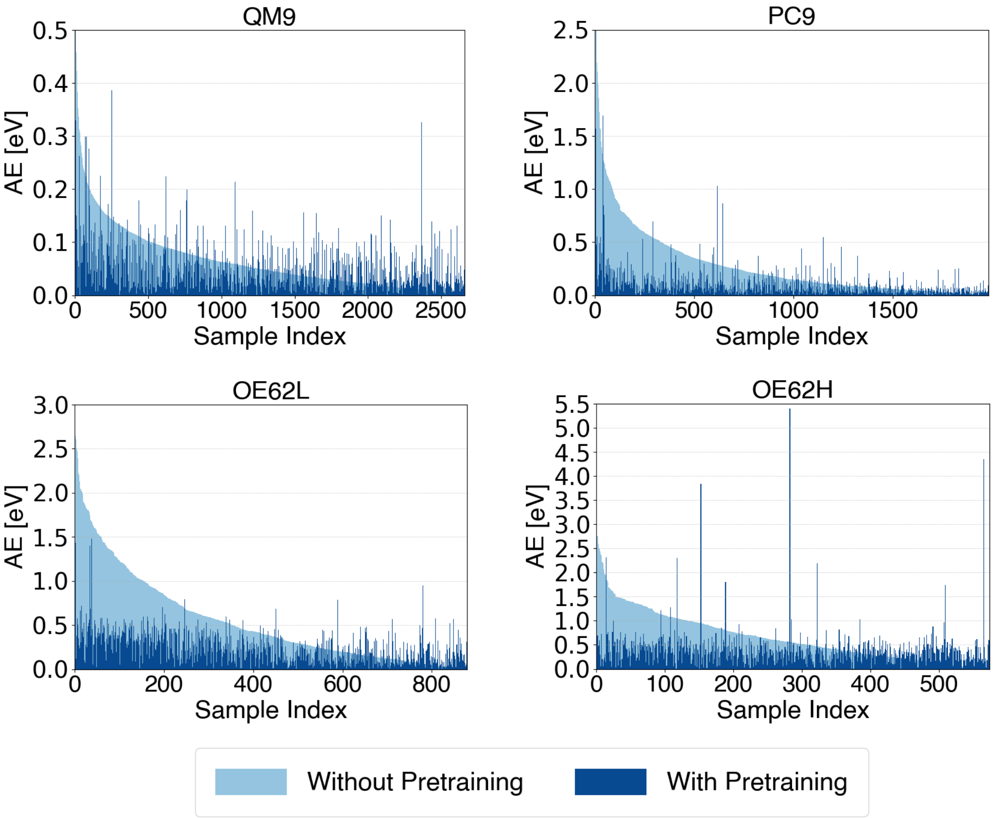

Figure S4: Per-sample absolute errors (AEs) of qs*GW* QP gap energy predictions from small models with and without pretraining.

Reduction of data demand in finetuning

We investigate if pretraining reduces the data demand in finetuning for qs*GW* QP LUMO energies gaps. For this purpose, we pretrain models on DFT LUMO energies and gaps and afterwards finetune on qs*GW* QP LUMO energies and gaps of 10 000, 20 000, 40 000, 80 000 and 120 000 finetuning samples respectively. Consistent with the analysis in the main text, we use three independently sampled sets for each number of finetuning samples to reduce the influence of small sample effects through averaging. Below in Fig. S5 and S6 we show the resulting learning curves for qs*GW* QP LUMO and gap predictions.

In line with the observation in the main text, we find that pretraining substantially reduces the amount of data needed in finetuning to converge the test losses for both LUMO energies and gaps,

especially on PC9 and OE62L. Like with HOMO energy predictions, in most cases roughly 40 000 finetuning samples thus one third of the full finetuning set is sufficient to achieve convergence of test losses. Sole outlier in this regard is the OE62H set where at least around 80 000 finetuning samples are required to reach MAE of 0.2 eV or lower. Also in consistency with the main text, we observe instabilities in the learning curves without pretraining, in particular on the OE62L set, which seems to stabilized by pretraining where we find monotonic decrease of test errors.

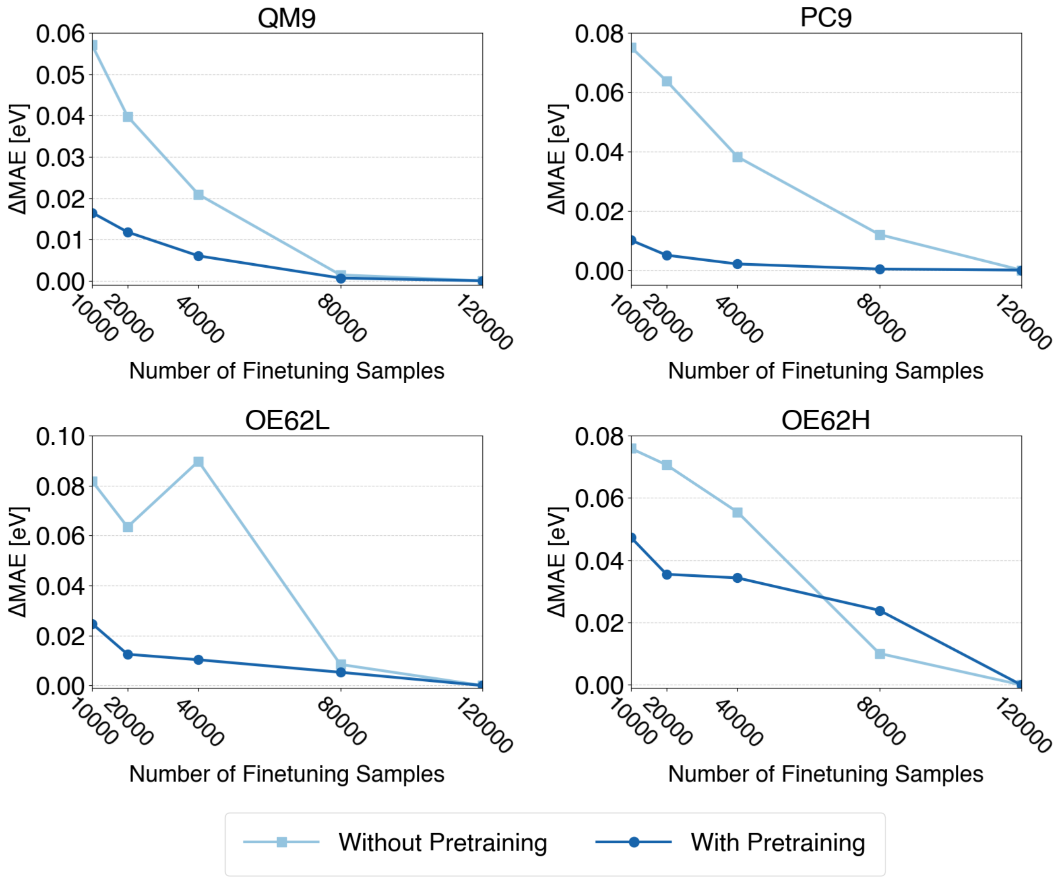

Figure S5: MAEs of QP LUMO energy predictions after finetuning on different numbers of samples with and without prior pretraining normalized to the respective MAE when finetuning on the full finetuning-set (120 000 samples).

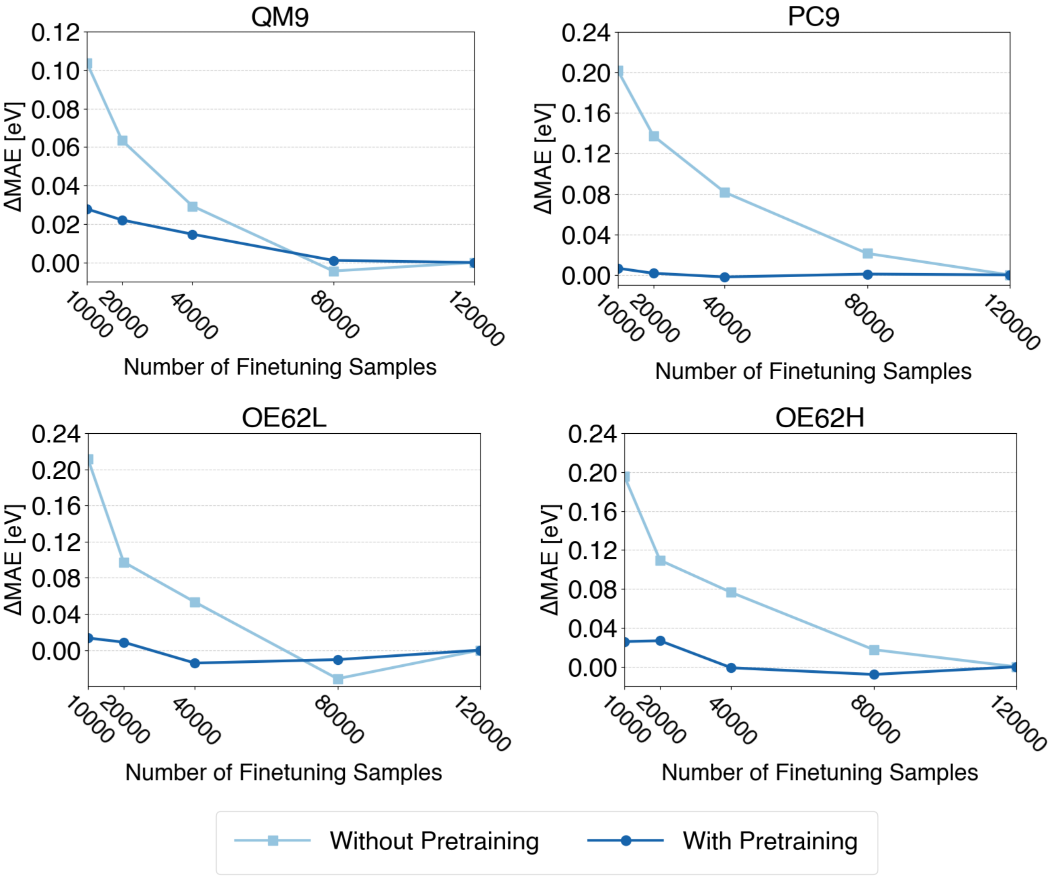

Figure S6: MAEs of QP gap energy predictions after finetuning on different numbers of samples with and without prior pretraining normalized to the respective MAE when finetuning on the full finetuning-set (120 000 samples).
